# Influence of Operating Temperature on Crack Growth Characteristics and Fatigue Life Prediction of Carbon Black-Filled Hydrogenated Nitrile Butadiene Rubber

**DOI:** 10.3390/polym16243574

**Published:** 2024-12-21

**Authors:** Runze Li, Zisheng Lian, Wensheng Wei

**Affiliations:** College of Mechanical Engineering, Taiyuan University of Technology, Taiyuan 030024, China; lirunze714@163.com (R.L.); weiwensheng2020@163.com (W.W.)

**Keywords:** fatigue crack growth, life prediction, temperature dependency, strain energy density, elastomer rubber

## Abstract

Rubber is widely used in situations involving cyclic loads, and the influence of temperature on rubber properties is particularly pronounced under cyclic loading. In this study, mechanical property tests and crack propagation tests of carbon black-filled hydrogenated nitrile butadiene rubber were conducted at four different operating temperatures. Based on the results of the crack propagation tests, the temperature-dependent characteristics of the Paris–Erdogan parameters and strain energy density were clarified. The Paris–Erdogan parameters were successfully expressed as a function of temperature. The strain energy density, on the other hand, exhibited the property of being strongly influenced by factors of strain, loading frequency, and others, while the temperature dependence was weak. On this basis, the unified fatigue crack growth kinetic model was constructed at multiple temperatures. The model results can match the experimental data well, particularly at temperatures of 60 °C and 80 °C. Finally, the fatigue life prediction model at different temperatures was constructed by combining the fatigue life test results. The results indicate a correlation between crack propagation characteristics and fatigue life predictions across different operating temperatures, with the predictions agreeing well with the measured life. The models can be used to analyze early fracture behavior or fatigue life prediction of rubber at different operating temperatures and minimize the need for extensive product testing prior to the manufacture of rubber products.

## 1. Introduction

Rubber materials, with their unique physical and chemical properties, have become an indispensable industrial raw material for the development of modern technology. Some of the most prevalent applications include tires, vibration dampers, packers, and water pressure artificial muscles, etc. [1,2,3]. Most of these components operate under cyclic loading, causing damage accumulation and ultimately leading to fatigue failure. Consequently, the investigation of the durability and fatigue resistance of rubber has become a prominent area of research in recent years [4,5,6]. The early prediction of rubber fatigue life has become a crucial objective in order to avert potential industrial hazards resulting from the fatigue failure of rubber in the aforementioned components [7,8,9,10].

Under cyclic loading, in addition to the influence of the working environment, the increase in the internal temperature of rubber is also influenced by self-heating, which cannot be ignored. The rise in temperature in this part, also known as “heat build-up” or “heat accumulation” [11], is caused by the hysteresis losses inherent to the loading and unloading cycles. It is acknowledged that higher temperatures can degrade the rubber, resulting in a reduction in fatigue life [12]. Building on the work of Mars [13], Gehling et al. [14] provided a more comprehensive and systematic overview of the key factors affecting fatigue behavior, including temperature, while taking into account overall developments and discoveries. As stated by [14], the fatigue life of styrene-butadiene rubber (SBR), a non-strain crystallizing rubber, decreases by a factor of 10^4^ under displacement control within the temperature range of 0 °C to 100 °C. On the contrary, the fatigue life of natural rubber (NR), a strain-crystallizing rubber, decreases only fourfold for the equivalent temperature change. Moreover, Ruellan et al. [15] demonstrated that temperature has an inhibitory effect on strain-induced crystallization (SIC). At temperatures up to 110 °C, even NR, which normally exhibits SIC, behaves as a non-strain crystallizing polymer, resulting in a deterioration of fatigue behavior. Barnabas Shaw et al. [16] investigated the effect of heat aging on the fatigue durability of hydrogenated nitrile butadiene rubber (HNBR) compounds. R. Stoček et al. [17] investigated the effect of the thermal aging behavior of rubber tires on their dynamic mechanical properties and fatigue crack propagation properties. The results show that the increase in aging temperature has a detrimental effect on crack growth resistance, which is manifested in all aged materials.

In contrast to the well-established fatigue theory for metallic materials, the fatigue life theory for rubber components is still under investigation [18,19]. Crack nucleation [20] and crack growth [21] are the two dominant modeling methods for predicting the fatigue life of rubber. Based on this, Luo et al. [22] and El Maanaoui et al. [23] developed models to predict the fatigue lifetime of carbon black-filled rubbers at different temperatures. El Yaagoubi et al. [24] employed the Monte Carlo principle to predict the lifetime of filled elastomers by combining the crack growth characteristics and agglomerate distribution within the component. It was demonstrated that the prediction curves exhibited a high degree of correlation with the observed lifetime under a range of testing conditions. Kocjan et al. [7] proposed a method to estimate operating temperatures and isothermal fatigue life curves based on experimental tests and numerical simulations of amorphous rubber compounds under fully relaxing uniaxial loading conditions. The model took into account the heat build-up effect and the influence of ambient temperature. Shangguan et al. [25] established a fatigue lifetime prediction model of filled natural rubbers based on the experimentally determined fatigue lifetime. Additionally, the effects of two important variables on life prediction were investigated: the specimen shape and the damage parameter.

Compared to the influence of loading frequency [7,14], the work focuses on the impact of operating temperature on the fatigue crack growth characteristics and life prediction of carbon black-filled HNBR. So, Based on the results of the crack propagation tests at multiple temperatures, the effect of temperature on fatigue crack propagation in rubber was analyzed. Additionally, the crack growth kinetic model and the fatigue prediction model at varying temperatures are established, and the life prediction model is verified with the fatigue life data. In the first section of the work, the materials and structures of the plane strain fatigue crack growth (FCG) test specimens employed in the fatigue crack growth test, as well as the dumbbell-type specimen used for the fatigue life test, are first presented. Next, all experimental research schemes are described in detail, including quasi-static uniaxial tensile tests for the development of a constitutive model of the material and the dynamic measurement methods, which are employed for the acquisition of the rubber crack growth characteristics and fatigue life. In Section 3, all measurement results are presented and discussed. Concurrently, the crack growth kinetic model and the fatigue lifetime prediction model at varying temperatures are developed in accordance with the Paris–Erdogan law. In the end, the principal results are summarized, while the potential factors that may be associated with the projected outcomes are analyzed.

## 2. Material and Methods

### 2.1. Material

In this study, samples of HNBR with a Shore A hardness of 60 were employed. The main components of the carbon black-filled elastomer formulation are shown in Table 1.

The vulcanization temperature was 175 °C, while the vulcanization pressure was found to be 10 MPa. The vulcanization time was determined to be 10 min. The samples measured 140 mm × 180 mm × 2 mm and were then subjected to secondary vulcanization in a hot air aging oven at 150 °C for four hours.

### 2.2. Quasi-Static Uniaxial Tensile Test

**Figure 1:**
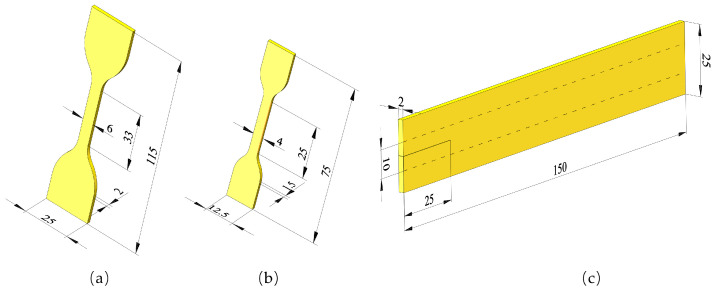
Specimen dimensions: (**a**) dumbbell-type specimen for uniaxial tensile test, (**b**) dumbbell-type specimen for fatigue life test, and (**c**) FCG test specimen for crack growth test.

Quasi-static uniaxial tensile (UT) tests were performed on the WDW-20 electronic universal testing machine (Changzhou Sanfeng Instrument Technology Corporation, Changzhou, China). The stress–strain characteristics of the specimen were measured sequentially at four different sets of temperatures. The tensile rate was 50 mm/min, and a non-contact laser extensometer was employed for the measurement of the nominal strain. The measurements at elevated temperatures were conducted through the use of a temperature-controlled chamber. Before testing, specimens were maintained in a temperature-controlled environment for approximately 30 min to ensure a uniform distribution of temperature throughout the specimen [26]. The dumbbell-type specimen employed in the test is shown in Figure 1a with accompanying dimensions in mm.

### 2.3. Fatigue Crack Growth Test

**Figure 2:**
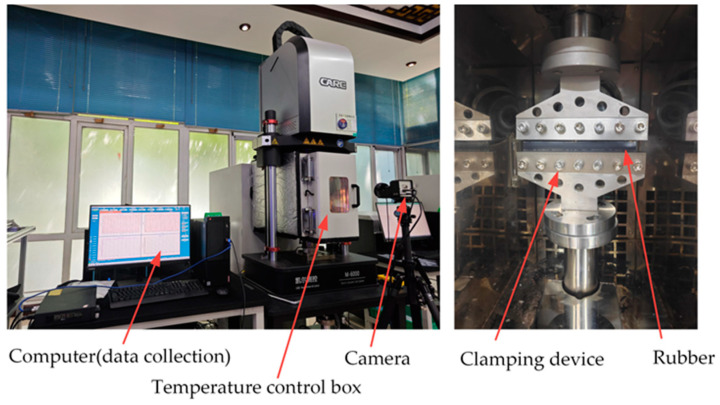
Dynamic fatigue testing machine and the specimen clamping method.

**Figure 3:**
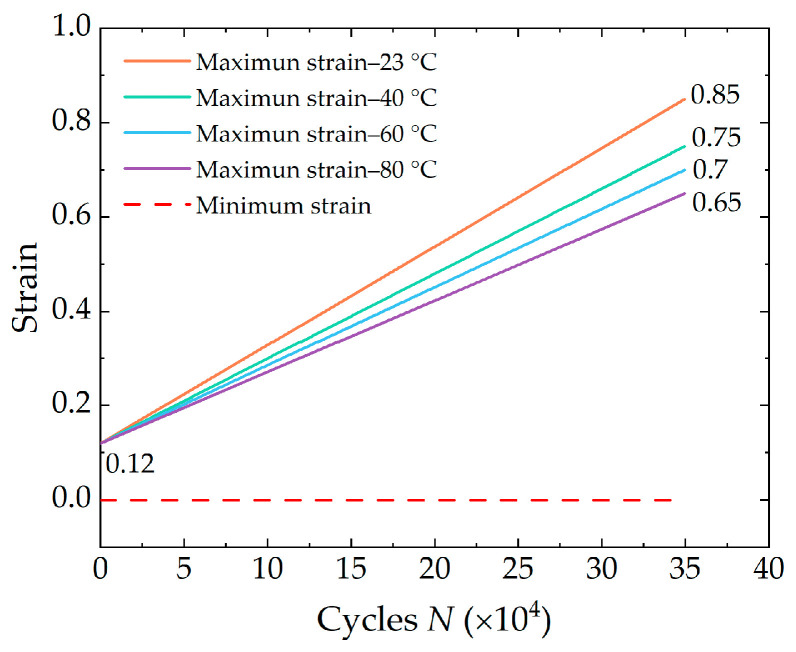
Strain levels of full relaxation variable amplitude cyclic loading at different temperatures.

The crack growth test was performed on the M6000 dynamic fatigue testing machine, (CARE Measurement & Control Test Systems Corporation, Tianjin, China) as shown in Figure 2. The relationship curves between the crack growth rate and the crack growth driving force were obtained by performing full relaxation cyclic loading tests on rubber specimens with prefabricated cracks. The crack growth characteristics of the specimens were measured sequentially at four different sets of temperatures. In this study, plane strain fatigue crack growth (FCG) test specimens were used, measuring 150 mm in length, 25 mm in height, and 2 mm in thickness (as illustrated in Figure 1c). The strain state of the specimen is not exactly identical to the pure shear strain state, and there is always some contraction along its length [27]. So, after the specimen was clamped on the fixture, the initial clamping height $h_{0}$ of the specimen was adjusted to a value of approximately 10 mm in order to meet the recommended minimum length-to-height ratio of greater than 10, thereby obtaining a longer pure shear crack propagation area. The initial crack was made by a smooth blade cut, as perpendicular to the loading direction as possible, with a length of 25 mm (greater than $2h_{0}$) to reduce the impact of the edge effect on crack propagation [28]. In order to obtain the rubber crack growth characteristics more rapidly and efficiently, a variable amplitude sinusoidal load with a loading frequency of 5 Hz was applied to the specimen. The maximum strain increased linearly from 12% to the corresponding preset values for each test temperature (as illustrated in Figure 3). A temperature-controlled chamber was used for the measurements at higher temperatures. The crack propagation tests were conducted at 5 Hz, chosen as an optimal compromise between the limitations of prolonged measurement time at low frequencies and the challenges of heat accumulation at higher frequencies.

During cyclic loading, the tearing energy was calculated by recording the stress value at each loading strain with the M6000 dynamic fatigue machine. The tearing energy T, which serves as the criterion for the mechanical fracture of rubber elastomers, can be derived from the energy balance during the crack growth. The tearing energy is formulated as:

(1) $$T=-\frac{\partial U}{\partial A}=-\frac{1}{t}\frac{\partial U}{\partial a}$$

where T is the tearing energy, U is the recoverable elastic strain energy, t is the thickness of the specimen, and a is the crack length.

With respect to the FCG test specimen, Rivlin and Thomas [29] demonstrated that the tearing energy T is independent of the crack length and can be defined as follows:

(2) $$T=W\cdot h_{0}$$

where W is the strain energy density stored in the specimen, and $h_{0}$ is the initial clamping height of the specimen in the zero-strain state.

As reported in several works [30,31], the definition of strain energy can be considered in terms of engineering stresses and strains. Therefore, the strain energy density of the FCG test specimen can be expressed as an equation for the force *F* and the displacement *s*, which can be defined as:

(3) $$W=\int \sigma \cdot d\varepsilon =\int \frac{F}{A_{uncr}}\cdot \frac{1}{h_{0}}\cdot ds=\frac{1}{A_{uncr}}\int F\cdot ds=\frac{U}{h_{0}A_{uncr}}$$

(4) $$A_{uncr}=t(l-a)$$

(5) $$T=\frac{U}{t(l-a)}$$

where $A_{uncr}$ is the uncracked area, l is the length of the specimen and the elastic strain energy U can be obtained by integrating the load–displacement curve in the fatigue crack extension test.

**Figure 4:**
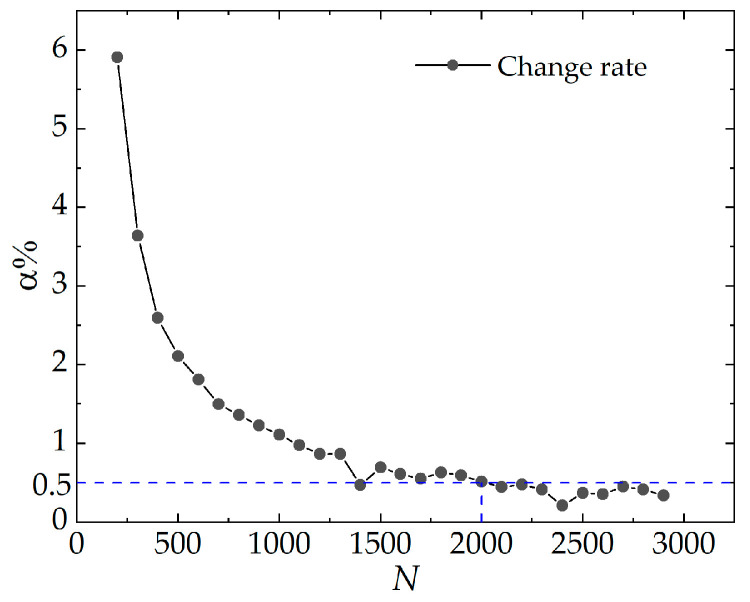
Relationship between the tearing energy change rate and precycles.

Prior to the crack growth tests, we conducted 3000 precycles on all unnotched specimens on the dynamic fatigue testing machine to eliminate the Mullins effect. Furthermore, the peak strain of precycles was determined to exceed the maximum strain value of the crack growth tests at each temperature. The tearing energy was monitored throughout each cycle, and the tearing energy change rate α% was calculated. The accommodation step was stopped when the value was less than 0.5 percent, indicating that the stress–strain response stabilized, as illustrated in Figure 4.

### 2.4. Fatigue Test

**Figure 5:**
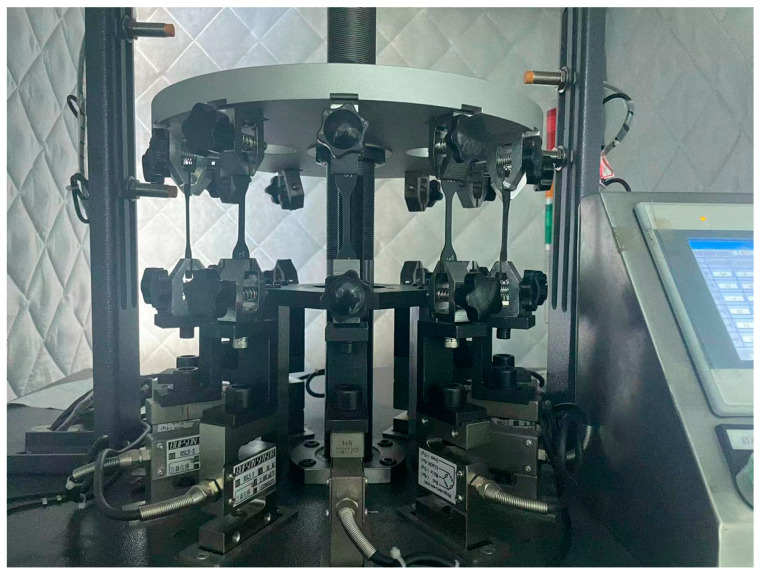
Installation of the specimen to the multi-station cyclic tensile fatigue testing machine.

The multi-station cyclic tensile fatigue machine (CARE Measurement & Control Test Systems Corporation, Tianjin, China) was used for the fatigue life test and specimens were installed on the testing machine through two metal clamps, as illustrated in Figure 5, with a peak engineering strain of 150%. Figure 1b illustrates the dimensions of the specimens employed in the fatigue test in mm. In consideration of the evident dissipative heating effect at high frequencies, the fatigue life test was conducted at 1 Hz to minimize the impact of heat accumulation on specimen life. To minimize the impact of the dispersion of rubber fatigue lifetime data on the test results [32], we have measured seven specimens simultaneously. Second, so as to eliminate the Mullins effect, each specimen was initially subjected to 2000 precycles at 200% engineering strain. Considering that the specimens would flex after pre-stretching, the peak engineering strain of the specimens after precycling was recalibrated to a predetermined value (150%), and the measurement was then started. As criteria for the fatigue measurement, the number of test cycles prior to the complete failure of the specimens was assumed to be indicative of the fatigue lifetime of the specimens.

## 3. Results and Discussions

### 3.1. Influence of Temperature on Mechanical Properties

**Figure 6:**
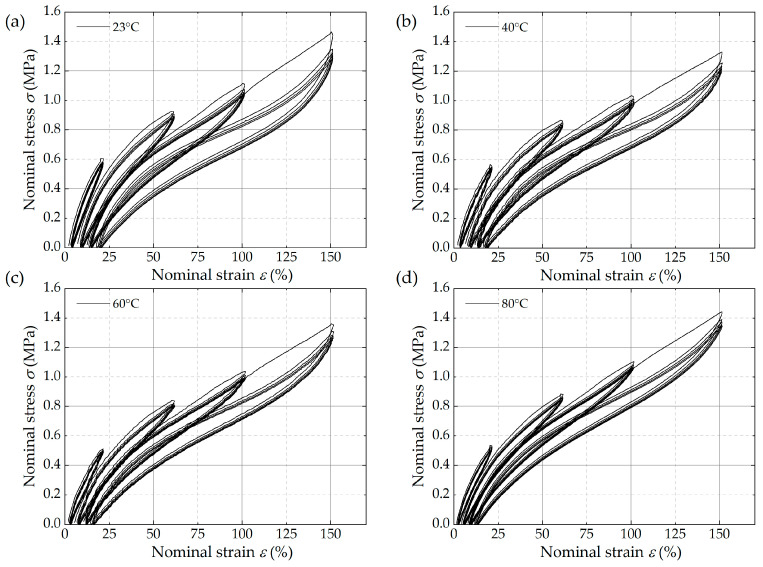
Uniaxial tensile stress–strain curves at four different operating temperatures: (**a**) 23 °C, (**b**) 40 °C, (**c**) 60 °C, and (**d**) 80 °C.

Figure 6 shows the stress–strain curves measured in uniaxial tensile tests at different operating temperatures, with five loading and unloading cycles at strain amplitudes of 20%, 60%, 100%, and 150%, respectively. As shown in the figure, the multiple hysteresis phenomenon serves to illustrate the significant properties of carbon black-filled HNBR. An increase in strain amplitude results in a reduction in the stiffness of the material at varying temperatures, a phenomenon known as material softening [33]. Repeating loading and unloading cycles at the same strain amplitude are observed to lead to a reduction in stress values, a phenomenon referred to as stress softening. The region enclosed by the loading and unloading curves is referred to as the hysteresis, which arises from the internal friction of the material. The hysteresis effect is observed to increase in response to an increase in the strain amplitude, resulting in a corresponding accumulation of heat within the material. The residual deformation of the unloading curve when the stress returns to zero is called permanent deformation, which increases in accordance with the strain amplitude. Meanwhile, an increase in temperature can result in a decrease in hysteresis area and permanent deformation. The reason why the stress peak at 80 °C is slightly higher than that at 60 °C may be due to the different trends of entropy and enthalpy-related energy contributions in rubber materials. During the process of testing temperature from 23 °C to 80 °C, the entropy-related energy contribution of HNBR showed a linear increase, while the enthalpy-related energy contribution showed a nonlinear decrease, resulting in a decrease and then an increase in rubber stress [34].

**Figure 7:**
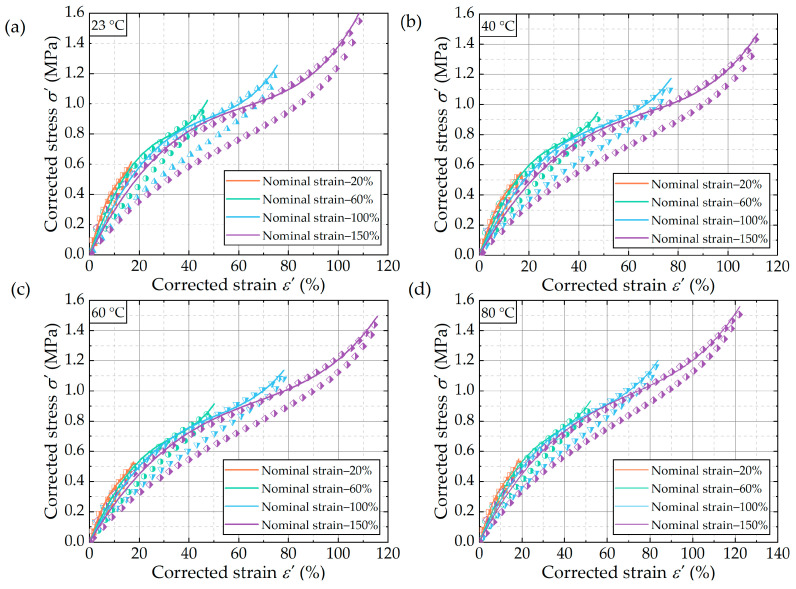
Fit curves of the stabilized cycles based on the Yeoh model for (**a**) uniaxial tensile at 23 °C, (**b**) uniaxial tensile at 40 °C, (**c**) uniaxial tensile at 60 °C, and (**d**) uniaxial tensile at 80 °C.

Figure 7 presents the fitting curve of the stable cycle of uniaxial tensile tests after four cycles of loading and unloading conducted at various test temperatures. Given the considerable permanent deformation of the rubber, the stress–strain data from the stabilization cycles were subjected to correction and subsequently fitted with the Yeoh model. The strain energy density under the model depends on the first invariant $I_{1}$ of the right Cauchy–Green deformation tensor, defined as follows:

(6) $$W=C_{10}(I_{1}-3)+C_{20}{\left(I_{1}-3\right)}^{2}+C_{30}{\left(I_{1}-3\right)}^{3}$$

where $C_{10}$, $C_{20}$, and $C_{30}$ are the material constants.

In this study, the data from the stable cycles of uniaxial tensile tests at different temperatures were fitted by Levenberg–Marquardt nonlinear least squares method, and the fitted parameters were obtained and listed in Table 2. As illustrated in Figure 7, the Yeoh model shows excellent fitting performance for uniaxial tensile tests at different temperatures for strains of 20%, 60%, 100%, and 150% (with correlation coefficients exceeding 0.99). It suggested that the Yeoh constitutive model is an effective approach for characterizing the stress–strain process of rubber at different temperatures.

### 3.2. Influence of Temperature on Fatigue Crack Growth Properties

According to the relationship between the tearing energy T and the fatigue crack growth (FCG) rate da/dN, the rubber crack growth curve can be roughly divided into four regions in the double logarithmic coordinate system [35,36]. In the first region, where the value of the tearing energy is below the threshold $T_{0}$, the crack grows slowly at a constant rate. In the second region, the FCG rate is observed to be a linear function of the tearing energy. As the tearing energy increases to the transition value $T_{t}$ (transient tearing energy), the crack growth enters the third region. In this region, the relationship between the FCG rate and the tearing energy changes to a power law relationship until the critical tearing energy $T_{c}$ is reached. Subsequently, the crack growth proceeds to the fourth region of the unstable fatigue growth stage, where the FCG rate tends towards infinity. The crack growth behavior represented by these four regions can be expressed by the following Equations:

(7) $$\mathrm{Stage}\;1:\;T<T_{0}\to da/dN=r\to 0$$

(8) $$\mathrm{Stage}\;2:\;T_{0}\leq T<T_{t}\to da/dN=A(T-T_{0})+r$$

(9) $$\mathrm{Stage}\;3:\;T_{t}\leq T<T_{c}\to da/dN=B\cdot T^{\beta }$$

(10) $$\mathrm{Stage}\;4:\;T_{c}\leq T\to da/dN\to \infty$$

where B and β are parameters related to the elastomer material, and the parameter β, as a crack growth exponent, reflects the intrinsic properties of the elastomer.

#### 3.2.1. Acquisition of the Crack Growth Lengths

As shown in Figure 3, the crack propagation test had 350,000 cycles, which can be divided into 700 stress levels and 500 reciprocating tensile cycles at each stress level. The camera was set to take a picture at the last FCG cycle of each stress level and was triggered to start taking pictures at the maximum displacement. Due to the testing frequency of 5 Hz, a picture was taken every 100 s.

Concurrently, the machine was equipped with a camera system; the CCD camera had 5 megapixels and was used to measure the actual crack lengths of the specimens at predetermined time steps. After obtaining photographs of the crack lengths of the specimens at each strain level, the photographs were processed using computer software (e.g., “Image J 1.54f”) [37]. The software calibrated the pixels on the photographs by the appropriate length scale (the initial crack length) captured in the image in order to obtain the actual length represented by each pixel. Based on this, the crack growth length was calculated and combined with the number of cycles to calculate the crack growth rate at each strain level. Meanwhile, in calculating the corresponding tearing energy, the value was the average value in the range of 450 to 500 cycles at each strain level.

**Figure 8:**
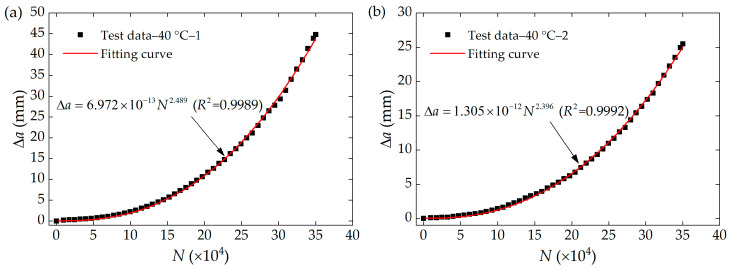
Crack growth length as a function of the number of fatigue cycles at 40 °C, (**a**) The first set of experiments, (**b**) The second set of experiments.

The relationship between the crack growth length Δa and the corresponding number of cycles N measured at 40 °C is shown in Figure 8. As illustrated in the figure, when the number of cycles was less than 100,000, the rate of crack growth was relatively slow. However, as the number of cycles exceeds 100,000, the FCG rate tended to accelerate apparently, as indicated by the increasing slope of the curve. The reason for the difference in the ultimate lengths of crack growth between the two sets of tests at 40 °C can be due to the different initial clamping heights $h_{0}$ of the specimens, as well as the differences in stress–strain curves at the same strain caused by prolonged fatigue cycles under crack propagation tests, ultimately resulting in the difference in the tearing energy values at the same strain. A power function was employed to establish the relationship between Δa and N throughout the entire process in both sets of tests, showing a strong correlation with coefficients exceeding 0.99. This favorable power-law characteristic was also evident in the FCG tests conducted at other temperatures.

**Figure 9:**
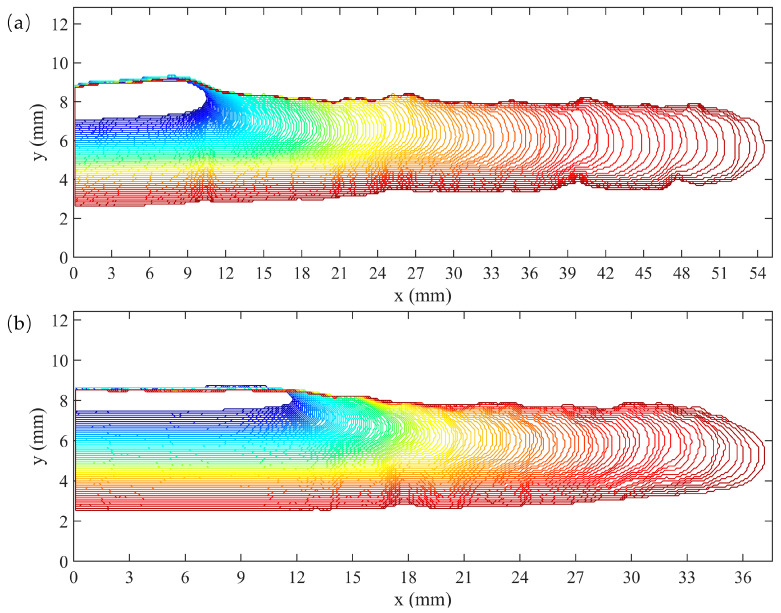
Crack profiles of the specimens in two sets of the crack propagation tests at 40 °C. x and y indicate the coordinate positions of the points forming the crack profiles, (**a**) The first set of tests, (**b**) The second set of tests.

Figure 9 illustrates the evolution of crack profiles in the specimens during the two sets of crack propagation tests at 40 °C as the number of cycles increases, with the *x*-axis representing the length direction of the specimen. It illustrated that during the initial phase of the FCG test, the crack began to propagate, and the crack tip gradually coarsened, forming a naturally rough crack. As the test proceeded, the crack tip invariably propagated along the direction perpendicular to the applied load, and no bifurcation phenomenon occurred throughout the entirety of the process. The crack growth rate also increased accordingly. Upon the completion of the loading cycle, the distance of the crack tip relative to the right free edge concerning the specimen exceeded 25 mm, thereby confirming that the observed crack growth was solely attributable to pure shear and not influenced by the free edge effect.

#### 3.2.2. Effect of Temperature on Crack Propagation Characteristics

**Figure 10:**
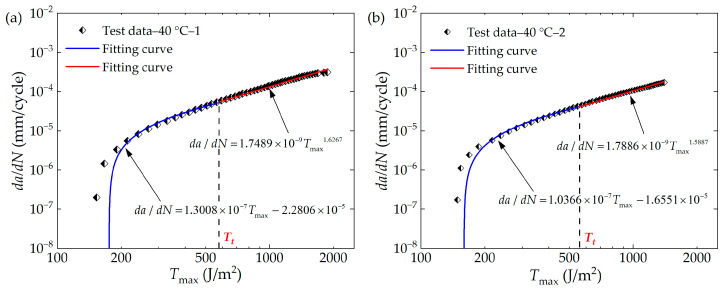
Crack growth rate as a function of the peak tearing energy at 40 °C (in the double logarithmic coordinate system), (**a**) The first set of experiments, (**b**) The second set of experiments.

It must be considered that both the traditional secant method and the seven-point polynomial method are prone to scattering and negative values in the calculation of crack growth rates subjected to variable amplitude loading conditions [38]. So, it is imperative to investigate alternative or refined methodologies to enhance accuracy and reliability. In this study, a power function was utilized to model the dependence of crack growth length Δa on the number of cycles N across different temperatures. Subsequently, the fitted power function was differentiated to determine the crack growth rate. The peak tearing energy $T_{\max}$ corresponding to crack growth length Δa was determined by Equation (5). Figure 10 shows the FCG rate as a function of the peak tearing energy at 40 °C, plotted on a double logarithmic scale. It clearly indicated that the rubber crack growth proceeded through the second and third stages. According to the nonlinear least squares method, it was found that the crack growth rate da/dN and the peak tearing energy $T_{\max}$ in the third stage, marked by the peak transition tearing energy $T_{t}$, adhere to the following functional relationship:

The first experiment:

(11) $$\mathrm{Stage}\;3:\;da/dN=1.7489\times 10^{-9}\cdot T_{\max}^{1.6267}\;(T_{t}=597{\;J/m}^{2};R^{2}=0.98)$$

The second experiment:

(12) $$\mathrm{Stage}\;3:\;da/dN=1.7886\times 10^{-9}\cdot T_{\max}^{1.5887}\;(T_{t}=567{\;J/m}^{2};R^{2}=0.99)$$

The validity of the experimental data was demonstrated by the high reproducibility observed in the results from the two sets of experiments. Application of the same method to the experimental results at other temperatures demonstrated strong agreement as well.

The subsequent section focuses on a detailed investigation into the influence of temperature on the behavior of elastomers during the third stage of crack propagation. The relationship between the mean crack growth rate da/dN and the peak tearing energy $T_{\max}$ was characterized by the Paris–Erdogan equation [28], resulting in the derivation of fitted curves. In addition, the Paris–Erdogan parameters B and β were determined at four sets of test temperatures.

**Figure 11:**
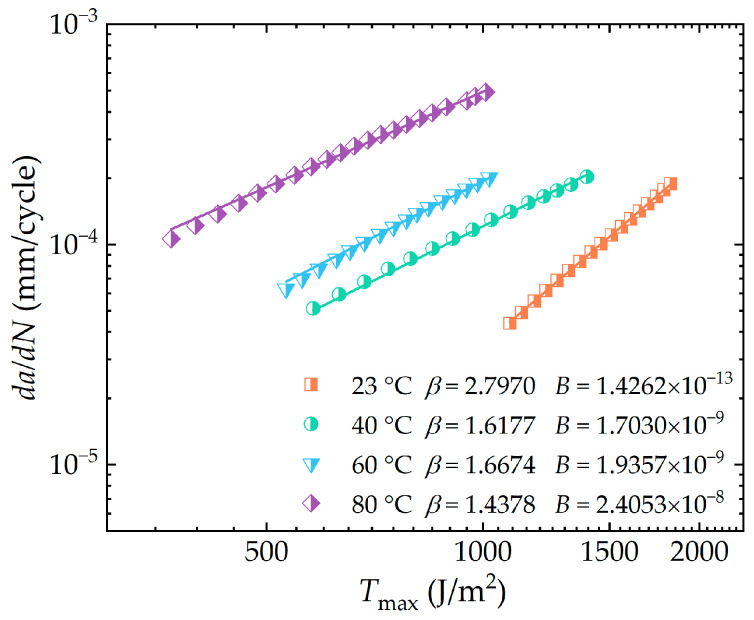
Crack growth rate as a function of peak tearing energy in the third stage of hydrogenated nitrile butadiene rubber (HNBR) elastomer at four temperature environments (in the double logarithmic coordinate system).

Figure 11 illustrates the double logarithmic representation of the peak tearing energy and the mean crack growth rate at different temperatures. The correlation coefficients between the fitted curves and the test data illustrated in the figure were all above 0.99. As shown in the figure, these curves clearly indicate that the tearing energy can play a decisive role in the propagation of rubber cracks. The rate of rubber crack growth was influenced by both the experimental temperature and the peak tearing energy value. An increase in peak tearing energy at a constant experimental temperature was accompanied by an increase in crack growth rate. Similarly, at a constant level of peak tearing energy, an increase in experimental temperature resulted in an acceleration of the rubber crack growth. A nearly tenfold increase in the crack growth rate could be observed when the temperature was increased from 23 °C to 80 °C under constant peak tearing energy conditions. Despite an equal increase in temperature, the changes in crack growth rate were more pronounced as the temperature increased from 23 °C to 40 °C and from 60 °C to 80 °C, compared to the shift between 40 °C and 60 °C. In terms of the fitted parameters β and B, the overall trend of the slope β tended to decrease with rising temperature, while the longitudinal intercept B showed an increase with increasing temperature. Similarly, the alteration in the fitted parameters from 40 °C to 60 °C was considerably less significant than the change from 23 °C to 40 °C. Meanwhile, the tearing energy transition value $T_{t}$ also decreased with increasing temperature, suggesting that higher temperatures can accelerate the progression of rubber crack propagation from the second to the third stage. It may be explained by the fact that at high temperatures, due to lower energy dissipation, more energy is available for crack propagation at the same tearing energy value, thereby accelerating the crack growth rate. Furthermore, the figure shows that the crack propagation index β for rubber at 23 °C is significantly higher compared to elevated temperatures. It was also observed that the crack propagation curve at 23 °C shifted towards a higher tearing energy range, suggesting an enhanced resistance to crack initiation while a decreased resistance to crack growth of the rubber.

#### 3.2.3. Extension of the Paris-Erdogan Law on Temperature Dependence

**Figure 12:**
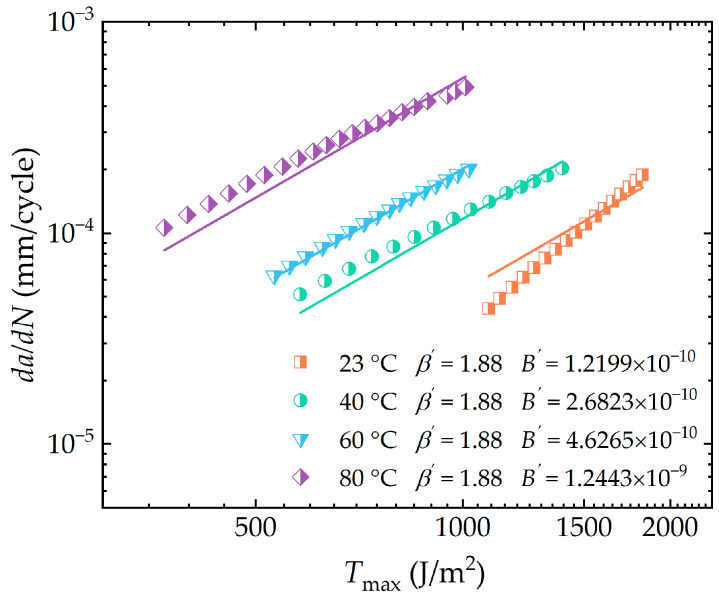
Fitting of crack growth rate with peak tearing energy under different temperature environments based on material parameters $\beta^{\prime }$ and $B^{\prime }$ (in the double logarithmic coordinate system).

To establish the dependence of crack growth rates on temperature, the Paris–Erdogan equation, which describes the characteristics of the third stage of crack growth, needs to be extended to incorporate temperature as a variable. In accordance with Equation (9), the parameter β is determined by the intrinsic properties of the rubber material, while the parameter B is designated as a function of temperature. Building on the parameters β at different temperatures shown in Figure 11, a new and unique material parameter $\beta^{\prime }$ was obtained using the least squares method. The parameters $B^{\prime }$ at different temperatures were derived by fitting the experimental data using the Paris–Erdogan equation with $\beta^{\prime }$ as the exponent. Figure 12 demonstrates a high degree of correlation between the experimental data and the fitted curves for HNBR material at four distinct measurement temperatures. As can be seen, power function curves based on the new parameter $\beta^{\prime }$ as an exponent, match well with the experimental results. This seems to suggest that the crack propagation behavior of the specimens at different temperatures is relatively similar. It can be reasonably concluded that the primary effect of temperature is to lower the energy barriers inside the rubber during crack propagation, which results in a parallel distribution of crack propagation curves at different temperatures.

**Figure 13:**
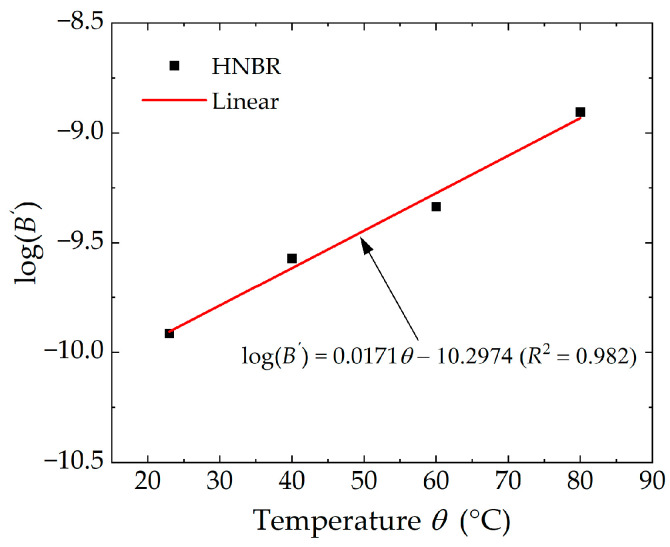
Fitted curve of material parameter $B^{\prime }$ with test temperature.

Based on the parameters $B^{\prime }$ at different temperatures shown in Figure 12, the linear function shown in Equation (14) was used to fit $\log(B^{\prime })$ to the test temperature θ. As illustrated in Figure 13, a strong linear correlation between $\log(B^{\prime })$ and θ is presented (with the correlation coefficient exceeding 0.98). So far, the temperature-dependent material parameter $B^{\prime }$ has been incorporated into the Paris–Erdogan law and the corresponding material parameter $\beta^{\prime }$ has been derived, enabling the Paris–Erdogan law to accurately predict the crack propagation behavior of HNBR rubber across varying temperatures.

(13) $$\log(da/dN)=\log B^{\prime }+\beta^{\prime }\log T$$

(14) $$\log B^{\prime }=b_{0}\theta +b_{1}$$

In light of the Paris–Erdogan law, the fatigue crack growth kinetic model at varying temperatures can be derived as follows:

(15) $$da/dN=10^{0.0171\theta -10.2974}T_{\max}^{1.88}$$

### 3.3. Analysis of Fatigue Test Results

**Figure 14:**
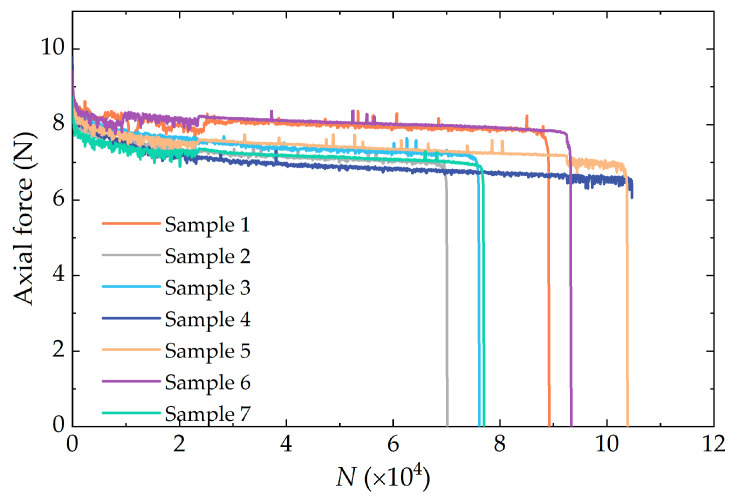
Axial force evolution with the number of cycles for seven dumbbell-type samples.

At a predetermined peak engineering strain, the relationship between the axial force of seven dumbbells and the number of cyclic loading cycles is presented in Figure 14. The analysis demonstrated that each dumbbell-type specimen, subjected to the same fatigue load, exhibited comparable behavioral characteristics. In the initial phase of cyclic loading, a pronounced decrease in the axial force of the specimens was observed. As the number of cycles increased gradually, the decline in the axial force of the specimens tended to stabilize until it abruptly plummeted to zero. At this juncture, the rubber specimen experienced fatigue fracture, and the corresponding number of cycles was defined as the fatigue life of the rubber specimen.

Research has indicated that the fatigue lifetime of rubber tends to follow a log-normal distribution at a given strain level [32]. Accordingly, based on the dispersion consideration of the fatigue test results, the following Equation was employed to calculate the logarithmic mean of the rubber fatigue lifetime at the 150% strain level. The measured data and the lifetime mean value under 150% strain level are presented in Table 3.

(16) $$\log_{10}(N_{ave})=\sum_{i=1}^{n}\log_{10}(N_{i})/n$$

where $N_{ave}$ is the average value of fatigue lifetime, n is the number of effective specimens for fatigue tests, and $N_{i}$ is the fatigue lifetime corresponding to the *i*-th dumbbell specimen.

## 4. Method of Fatigue Lifetime Prediction

### 4.1. Verification of Temperature-Based Fatigue Crack Growth Model

To verify the accuracy of the unified model for fatigue crack growth at multiple temperatures based on the Paris–Erdogan law in Section 3.2, We have predicted the fatigue life of a dumbbell-type specimen under 150% strain load and compared it with the fatigue life test results.

The tearing energy as a function of the strain energy density for a single-edge notched dumbbell-type specimen is defined as follows:

(17) T=2kWa

where a is the crack length, k is a factor related to the strain level of the specimen and can be defined as follows [39]:

(18) $$k=\frac{2.95-0.08\varepsilon_{\max}}{{\left(1+\varepsilon_{\max}\right)}^{1/2}}$$

Substituting Equations (17) and (18) into the fatigue crack propagation model represented by Equation (15) and integrating Equation (15), the fatigue lifetime calculation formula for rubber materials under uniaxial tensile fatigue loading can be defined as:

(19) $$N_{f}=\frac{1}{B(\beta -1){\left(2kW\right)}^{\beta }}(a_{0}^{1-\beta }-a_{f}^{1-\beta })$$

where $a_{0}$ is the initial crack length of the HNBR test material, $a_{f}$ is the maximum crack length corresponding to the sample undergoing fatigue fracture.

The initial defect sizes observed in rubber materials are typically distributed within the range of 20 µm to 60 µm [40]. In this study, the uniaxial tensile fatigue lifetime of dumbbell-type specimens with different initial crack sizes was determined using Equation (19), with the values of $a_{0}$ taken as 20 µm, 30 µm, and 40 µm, respectively. Furthermore, determining the fatigue life of the specimen necessitates the application of a criterion to define when a failure occurs. The majority of preceding studies have employed two methods to determine fatigue life: the fatigue crack length failure criterion and the stiffness-based failure criterion [41]. Harbour et al. investigated the difference in fatigue life between NR and SBR materials under two criteria. It was demonstrated that, with a critical crack length of 1 mm, the predicted fatigue life based on the crack length failure criterion tended to be more conservative for both materials [42]. Consequently, a critical crack length of 1 mm was employed in the calculation of the fatigue life.

The predicted fatigue life of dumbbell-type specimens, based on three initial crack lengths at a tensile strain of 150%, is presented in Table 4. As can be seen from Table 4, the fatigue life results are significantly influenced by the initial crack size. When $a_{0}=20$ µm, the ratio of the predicted fatigue life result $N_{p}$ to the mean value $N_{ave}$ of the test results was approximately one, thus corroborating the accuracy of the derived crack growth model. Concurrently, the average value of the measured fatigue life results could be incorporated into Equation (19) to determine the actual initial crack size of the rubber material, $a_{0}=22.83$ µm.

### 4.2. Fatigue Life Prediction at High Temperature

In the previous section, the fatigue life model of rubber materials under uniaxial tensile fatigue loading at room temperature was validated. In order to predict the fatigue life of rubber materials at multiple temperatures, it is essential to investigate the dependence of the W on both temperature and strain.

#### 4.2.1. Effect of Temperature on Strain Energy Density

**Figure 15:**
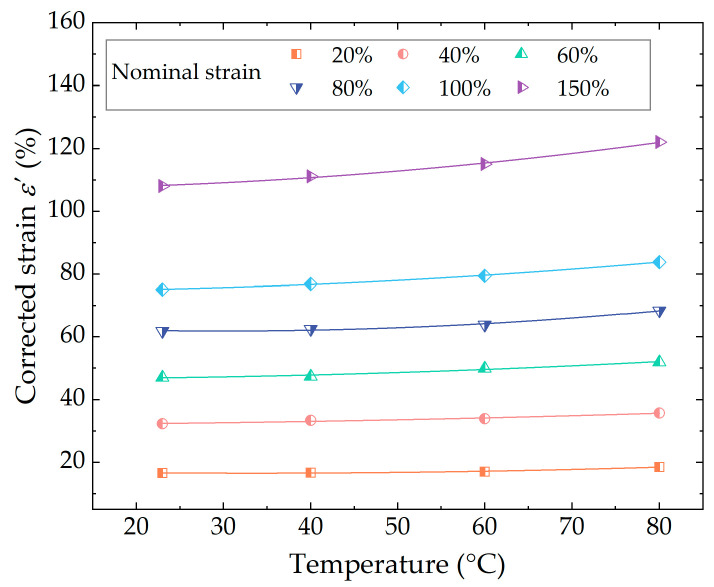
The relationship between corrected strain and test temperature.

So, the Yeoh model was used to fit the unloading segment of the stable cycle curve shown in Figure 7, and the corresponding fitting parameters were obtained. Subsequently, the dependence of parameters $C_{10}$, $C_{20}$, and $C_{30}$ on temperature was analyzed for each strain. Based on the results, the stress–strain curve at the corresponding strain was formulated as a function of temperature. Therefore, the value of W at different temperatures can be obtained by integrating the corrected stress–strain curve. It is worth noting that the corrected strain value $\varepsilon^{\prime }$, which represented the upper limit of integration, was also affected by temperature. The results of the polynomial fitting of the corrected strain $\varepsilon^{\prime }$ in relation to the temperature of the rubber material under uniaxial tensile fatigue loading are illustrated in Figure 15. As a result, the strain energy density as a function of both temperature and strain is calculated as follows:

(20) $$\begin{array}{l} W(\lambda ,T)=C_{10}(T)\cdot (\lambda^{2}+2\lambda^{-1}-3)+C_{20}(T)\cdot (\lambda^{4}-6\lambda^{2}+4\lambda -12\lambda^{-1}+4\lambda^{-2}+9)\\ +C_{30}(T)\cdot (\lambda^{6}-9\lambda^{4}+6\lambda^{3}+27\lambda^{2}-36\lambda +54\lambda^{-1}-36\lambda^{-2}+8\lambda^{-3}-15) \end{array}$$

where λ is the elongation, defined as: $\lambda =1+\varepsilon^{\prime }$.

**Figure 16:**
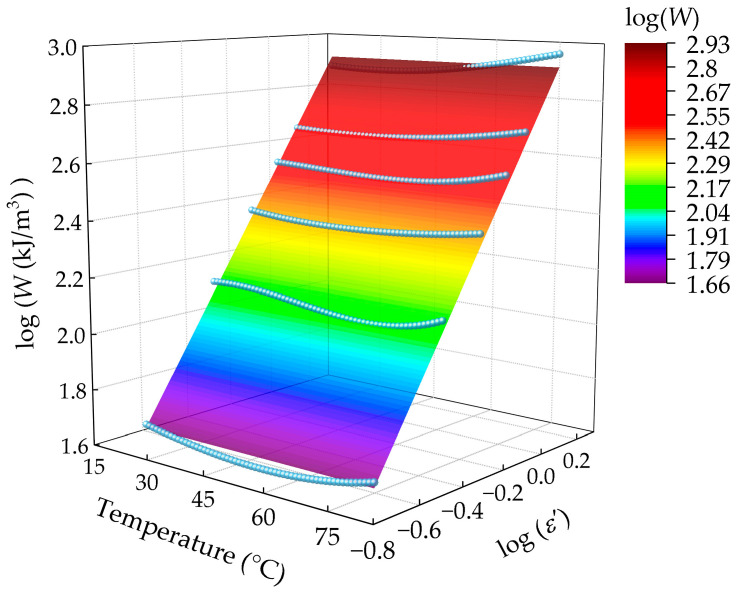
Relationship between logarithmic strain energy density values and logarithmic strain values in uniaxial tensile tests at different temperatures.

**Figure 17:**
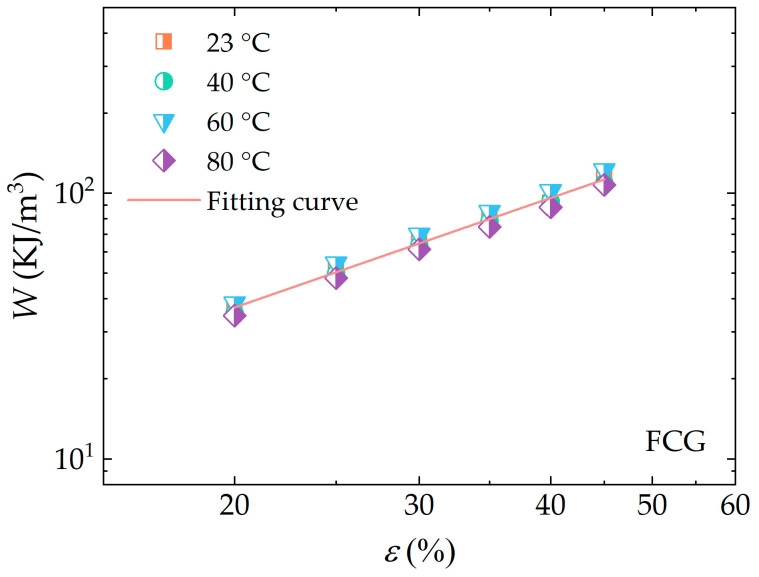
Measured data of strain energy density with test strain for crack growth tests at different temperatures (in the double logarithmic coordinate system).

Figure 16 shows the variation of logarithmic strain energy density values of dumbbell-type specimens in uniaxial tensile tests with respect to test temperature and logarithmic strain values. From the figure, it could be observed that when the strain values are equal, the values of W at different temperatures were almost equal, indicating a weak dependence of the strain energy density on temperature. Figure 17 illustrates the strain energy density distribution of FCG test specimens for crack propagation tests conducted at varying temperatures, once again substantiating this conclusion. These findings were also shown in [22]. As illustrated in Figure 17, the values of W and the maximum strain show a linear relationship in a double logarithmic coordinate system, defined as follows:

(21) $$W(\varepsilon )=336.7\times \varepsilon^{1.37}$$

Combining Equations (2), (15), and (21), the kinetic model for fatigue crack growth of carbon black-filled HNBR at different temperatures can be defined as:

(22) $$\frac{da(\varepsilon )}{dN}=10^{0.0171\theta -10.2974}\cdot {\left(336.7\cdot \varepsilon^{1.37}\cdot h_{0}\right)}^{1.88}$$

**Figure 18:**
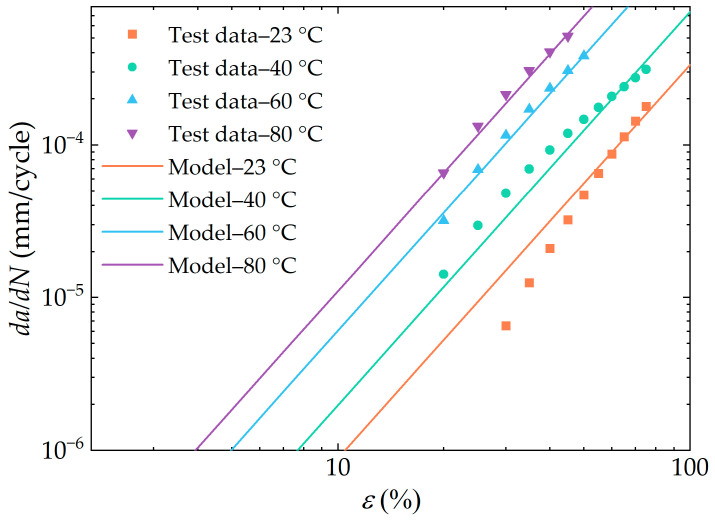
Fatigue crack growth rate prediction model (linear) and measured values (symbol) at multiple temperatures (in the double logarithmic coordinate system).

Figure 18 depicts the fatigue crack growth rate prediction model at multiple temperatures, derived from Equation (22), in comparison with the corresponding experimentally obtained data. The crack growth rate prediction model demonstrated a high degree of accuracy in matching the measured data, particularly at temperatures of 60 °C and 80 °C.

**Figure 19:**
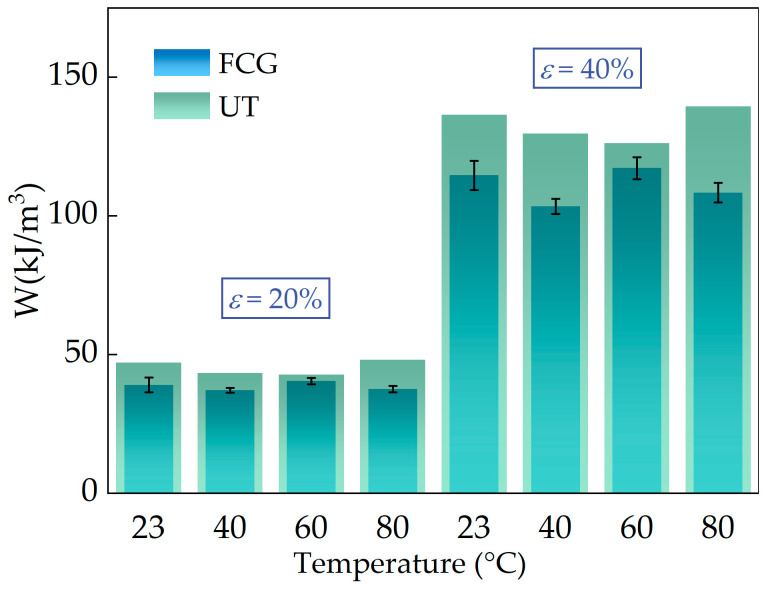
Strain energy density values for uniaxial tensile and crack growth tests at 20% and 40% strain values.

The values of W at various operating temperatures, corresponding to both types of tests conducted at 20% and 40% strain, are presented in Figure 19. The strain energy density values of FCG test specimens were observed to be less than those of dumbbell-type specimens at the same strain for all four temperatures measured. The reasons for this can be attributed to the higher frequency of crack propagation tests, which results in greater energy dissipation at the same strain, as well as greater permanent deformation caused by long reciprocating cyclic loading.

In conclusion, the strain energy density is significantly affected by factors such as strain, loading frequency, and others, while the temperature dependence is weak.

#### 4.2.2. Unified Model for Fatigue Life Prediction at Multiple Temperatures

Figure 16 illustrates a strong linear correlation between the logarithmic values of W and the logarithmic values of maximum strain in uniaxial tensile tests conducted at four distinct temperatures, which satisfies the power function relationship defined as:

(23) $$W=469.5174\times \varepsilon_{\max}^{1.3587}$$

In consequence, the fatigue life prediction model of rubber material under uniaxial tensile fatigue loading at high temperatures is defined as follows:

(24) $$N_{f}=\frac{1}{B(\theta )(\beta^{\prime }-1){\left(2kW(\varepsilon_{\max})\right)}^{\beta^{\prime }}}(a_{0}^{1-\beta^{\prime }}-a_{f}^{1-\beta^{\prime }})$$

where $a_{0}=0.02283\;\mathrm{mm}$, $a_{f}=1\;\mathrm{mm}$, $B(\theta )=10^{0.0171\theta -10.2974}$, $\beta^{\prime }=1.88$, $k=\frac{2.95-0.08\varepsilon_{\max}}{{\left(1+\varepsilon_{\max}\right)}^{1/2}}$, $W=469.5174\times \varepsilon_{\max}^{1.3587}{\;\mathrm{KJ}/m}^{3}$.

**Figure 20:**
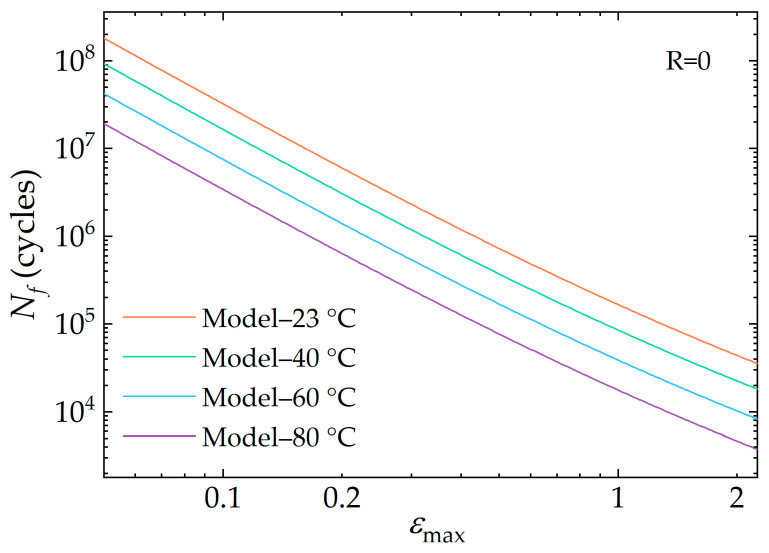
Relationship between maximum strain values and fatigue life at different temperatures (in the double logarithmic coordinate system).

The variation of the fatigue life prediction value with peak strain at varying temperatures is shown in Figure 20, which indicates that both temperature and strain are negatively correlated with the fatigue life of rubber materials. Furthermore, the relationship between fatigue life and maximum strain is no longer purely linear in the double logarithmic coordinate system, as the influence of the variation of k with strain $\varepsilon_{\max}$ has been considered.

## 5. Conclusions

In this study, the effects of experimental temperature on the stress–strain characteristics, crack growth rate, and fatigue life of rubber were analyzed. The fatigue crack growth kinetic model and the fatigue life prediction model at different temperatures were constructed based on the results of the uniaxial tensile test, the crack propagation test, and the fatigue life test.

The stress–strain data from the steady cycles of the uniaxial tensile tests were corrected and fitted using the Yeoh model. The measured multi-hysteresis properties indicate that an increase in temperature results in a reduction in both dissipation and permanent deformation of the material. In the study of crack propagation experiments, the temperature-dependent characteristics of the Paris–Erdogan parameters and strain energy density were investigated. An extension of the Paris–Erdogon law was achieved by expressing the Paris–Erdogon parameters as a function of temperature, making it possible to predict FCG rates for elastomers at different temperatures other than the test temperature. The strain energy density, on the other hand, exhibited the property of being strongly influenced by factors strain, loading frequency, and others, while the temperature dependence was weak. The results of crack propagation studies and fatigue predictions conducted at varying operating temperatures indicate a correlation effect. Under an identical strain amplitude, an increase in operating temperature could accelerate the crack growth rate, resulting in a shortened fatigue life. Furthermore, an increase in operating temperature from 23 °C to 40 °C had a considerably stronger effect than an equivalent rise from 40 °C to 60 °C.

The unified model developed in this study for predicting the fatigue life of rubber at various temperatures can effectively match the measured data, thus offering a viable approach for predicting the life of rubber components at different stress states and different operating temperatures during the engineering design process. Given that the Paris–Erdogan parameters have been expressed as a function of temperature. It is only necessary to obtain the strain energy density (cracking energy density) at room temperature under actual loading conditions, and then the model can be used to analyze the early fracture behavior or fatigue life prediction of rubbers at different operating temperatures. This reduces product development time and minimizes the need for extensive product testing prior to the manufacture of rubber products.

## 6. Limitations and Prospects

In the process of studying the influence of working temperature on the crack propagation characteristics of carbon black-filled hydrogenated nitrile butadiene rubber, the following shortcomings exist:

(1) The research material selected in the article was carbon black-filled HNBR, but due to the limited experimental conditions, the rest of the control group was not set up. The effect of the carbon black content was not considered. In future research, the characteristics of fatigue crack propagation of HNBR under the two variables of carbon black content and temperature can be investigated to make the research results more comprehensive.
(2) This manuscript predicted the fatigue life of rubber specimens under uniaxial tensile tests based on a unified model of fatigue crack growth and compared it with the test results. However, life prediction under multiaxial loading conditions was not addressed. Under simple or planar stretching, the energy release rate can be derived directly from the strain energy density, and then the life prediction can be solved by Equation (24). However, the fatigue life of rubber under multiaxial stress states requires the use of cracking energy density [43] as a fatigue life prediction parameter under multiaxial strain history. This parameter represents the available energy density for a given strain state and crack orientation.

Studying the life prediction of rubbers under different loading conditions, formulations, and temperatures is the focus of future research, which will greatly enhance the adequacy and practical relevance of the research. In addition, microstructural analysis of rubber will be more conducive to the study of various properties of rubber materials.

## Figures and Tables

**Table 1:** Recipe of the HNBR.

Component	Description	Phr
Hydrogenated nitrile butadiene rubber	HNBR	100
Zinc oxide	ZNO	10
Stearic acid	SA	1
plasticizer	TOTM	12
Antioxidant	MBZ	1
Antioxidant	445	1
Vulcanization accelerator	TAIC	2.5
Carbon black	N220	20
Carbon black	N990	25

**Table 2:** Fitting parameters of the Yeoh model at different temperatures.

Operating temperature 23 °C				
Strain level	$C_{10}$ (MPa)	$C_{20}$ (MPa)	$C_{30}$ (MPa)	$R^{2}$
20%	1.0198	−4.5256	21.5417	0.9915
60%	0.7583	−0.6389	0.5034	0.9898
100%	0.6226	−0.2372	0.0886	0.9889
150%	0.5445	−0.1243	0.02894	0.9885
Operating temperature 40 °C				
Strain level	$C_{10}$ (MPa)	$C_{20}$ (MPa)	$C_{30}$ (MPa)	$R^{2}$
20%	0.8962	−3.6039	16.6720	0.9907
60%	0.6935	−0.5476	0.4176	0.9895
100%	0.5727	−0.2028	0.0718	0.9894
150%	0.4920	−0.0972	0.0208	0.9914
Operating temperature 60 °C				
Strain level	$C_{10}$ (MPa)	$C_{20}$ (MPa)	$C_{30}$ (MPa)	$R^{2}$
20%	0.7968	−3.0062	13.6796	0.9864
60%	0.6058	−0.4022	0.2891	0.9941
Operating temperature 60 °C				
Strain level	$C_{10}$ (MPa)	$C_{20}$ (MPa)	$C_{30}$ (MPa)	$R^{2}$
100%	0.5273	−0.1642	0.0569	0.9916
150%	0.4603	−0.0778	0.0163	0.9938
Operating temperature 80 °C				
Strain level	$C_{10}$ (MPa)	$C_{20}$ (MPa)	$C_{30}$ (MPa)	$R^{2}$
20%	0.7906	−2.9751	13.3718	0.9953
60%	0.5918	−0.3441	0.2281	0.9949
100%	0.5113	−0.1337	0.0420	0.9945
150%	0.4587	−0.0670	0.0128	0.9944

**Table 3:** Uniaxial tensile fatigue test results of dumbbell-shaped specimens.

**Peak Strain**	$N_{1}$	$N_{2}$	$N_{3}$	$N_{5}$	$N_{6}$	$N_{7}$	$N_{ave}$	$\frac{N_{\max}}{N_{\min}}$
150%	89,078	69,743	75,848	103,431	92,455	76,568	83,747	1.483

**Table 4:** Comparison between predicted and measured uniaxial tensile fatigue life of dumbbell-type specimens with different initial crack sizes.

Peak Strain	$N_{ave}$	$a_{0}\;(m)$	$N_{p}$	$N_{p}/N_{ave}$
150%	83,747	$2\times 10^{-5}$	94,478	1.128
		$3\times 10^{-5}$	49,911	0.596
		$4\times 10^{-5}$	33,996	0.406

## Data Availability

The authors declare that the main data supporting the findings and conclusions of this study are available within the article. Original and additional data is available from the corresponding author upon request.
